# The International Encyclopædia of Surgery

**Published:** 1886-09

**Authors:** 


### Book Reviews.

---

The International Encyclopædia of Surgery. A Systematic Treatise on the Theory and Practice of Surgery by Authors of various Nations. Edited by John Ashhurst, Jr., M. D., Professor of Clinical Surgery in the University of Pennsylvania. Illustrated with chromo-lithographs and wood-cuts. In six volumes. Vol. VI. New York: Wm. Wood & Company. 1886.

This is the sixth and last volume of the encyclopædia, and is fully equal to the preceding ones.

Professor J. Solis-Cohen leads off with forty-four pages on Injuries and Diseases of the Œsophagus in the plain, easy style for which he is noted. The article is profusely illustrated, which adds much to its value.

Intestinal Obstruction, by John Ashhurst, Jr., M. D., Professor of Clinical Surgery in the University of Pennsylvania, forms the second article.

The author divides intestinal obstruction into acute and chronic. He discusses at some length the various causes of the different forms. In the treatment the writer says there is probably no class of cases so habitually mismanaged by otherwise intelligent practitioners as cases of intestinal obstruction. He condemns the reckless administration of drugs in these cases as not only doing no good, but actual harm. He warns against the hasty resort to operative measures encouraged by the much-vaunted modern triumphs of "abdominal surgery," believing that many lives might be saved by more rational if less brilliant treatment. He says:

"I speak strongly upon this subject, because I feel a certain degree of personal responsibility in the matter, an article published by myself some twelve years ago having had a share in popularizing the operation of abdominal section in these cases. I would strongly urge that the surgeon should not, in any case of intestinal obstruction, open the abdomen, unless he has been able to form some distinct notion of what he expects to find, or at least until he has been able to satisfy himself that there is positive mechanical occlusion, and that no less dangerous operation will suffice for its relief."

He regards most cases of acute intestinal obstruction met with in practice as cases of enteritis, and recommends bloodletting, either locally by means of leeches, or, if the subject be a vigorous adult, general bloodletting. He advises, after the bleeding, that the abdomen be covered over with mercurial oitment and a warm mush or hop poultice applied over this. The patient should be brought speedily under the influence of opium, and warm enemata of water or oil may be employed. He advises against purgatives given by the mouth. If the symptoms do not subside blisters may be used to advantage. This treatment relates only to those cases not caused by mechanical obstruction.

The article closes with a table containing references to 186 cases of enterectomy and colectomy. Of these 186 cases 93 are reported as recoveries and 86 deaths; the result in seven cases was not known.

Injuries and Diseases of the Rectum, by William Allingham, F. R. C. S., London, forms one of the most interesting chapters in this work.

E. L. Keys, A. M., M. D., Professor of Genito-Urinary Diseases in the Bellevue Hospital Medical College, follows in a paper, covering 155 pages, on Urinary Calculus. On the subject of geographical distribution of stone, the writer says: "In North

America, in the United States, the greatest number of stone cases originate in central districts—Tennessee, Kentucky, Ohio, Indiana, Missouri, Western Pennsylvania and Virginia. In the Northern, Eastern, the Gulf, Southern and Western States calculus is uncommon, as it also is in the Canadas and British possessions." These peculiarities of distribution are unaccounted for.

The paper is well illustrated by chromo-lithographs illustrating the appearances of some of the forms of urinary calculus.

Lithotrity, by Wm. H. Hinston, M. D., Professor of Clinical Surgery in the Montreal School of Medicine, forms an interesting paper.

Following this we have injuries and diseases of the bladder and prostate, by Reginald Harrison, F. R. C. S., Lecturer on Clinical Surgery in the Victoria University, Liverpool; Injuries and diseases of the urethra, by Simon Duplay, M. D., Professor of Clinical Surgery in the Faculty of Medicine of Paris.

On the subject of treatment of strictures he says: "Among the different methods of treating strictures, it is to inflammatory atrophic dilatation that the first rank should without contradiction be accorded. It is the most simple, the easiest to carry out, the most exempt from danger, finally which gives the most durable results, especially if care be taken to continue the employment of it even when patient appears to be cured. It is to it, then, that recourse should always be had by preference.

We think this is generally true of strictures of the deep urethra, but in those of the penile portion we have found gradual dilatation useless. Divulsion or internal urethrotomy must be resorted to in these cases. H. Rayes Bell, F. R. C. S., Surgeon to and Demonstrator of Surgery at Kings College Hospital, London, writes an able article on injuries and diseases of the male genital organs.

Theophilus Parvin, M. D., Professor of Obstetrics, Jefferson

Medical College, contributes the chapter on injuries and diseases of the female genitals.

The Cæsarean Section and its substitutes is written by Robert P. Harris, A. M., M. D., of Philadelphia.

Ovarian and uterine tumors, by Charles Carroll Lee, M. D., Surgeon to the Woman's Hospital, New York, gives a clear and concise history of those tumors with the different methods of treatment.

Then follows inflammatory affections of the bones, by L. Ollier, M. D., Professor of Clinical Surgery in the University of Lyons. Scrofulo-tuberculosis and other structural diseases of bones is by Eugene Vincent, M. D., of Lyons. Tumors of the bones is also by a surgeon of Lyons, A. Paucet. Orthopædic Surgery is by Frederic R. Fisher, F. R. C. S., London.

The volume ends with an elaborate general index which adds very much to the value of the work.

